# Hypofractionated intensity modulated irradiation for localized prostate cancer, results from a phase I/II feasibility study

**DOI:** 10.1186/1748-717X-2-29

**Published:** 2007-08-08

**Authors:** Sara Junius, Karin Haustermans, Barbara Bussels, Raymond Oyen, Bianca Vanstraelen, Tom Depuydt, Jan Verstraete, Steven Joniau, Hendrik Van Poppel

**Affiliations:** 1 Radiation Oncology, University Hospital Gasthuisberg, Herestraat 49, 3000 Leuven, Belgium; 2 Radiation Oncology, H. Hartziekenhuis, Wilgenstraat 2, 8800 Roeselare, Belgium; 3 Radiology, University Hospital Gasthuisberg, Herestraat 49, 3000 Leuven, Belgium; 4 Physics, University Hospital Gasthuisberg, Herestraat 49, 3000 Leuven, Belgium; 5 Urology, University Hospital Gasthuisberg, Herestraat 49, 3000 Leuven, Belgium

## Abstract

**Background:**

To assess acute (primary endpoint) and late toxicity, quality of life (QOL), biochemical or clinical failure (secondary endpoints) of a hypofractionated IMRT schedule for prostate cancer (PC).

**Methods:**

38 men with localized PC received 66 Gy (2.64 Gy) to prostate,2 Gy to seminal vesicles (50 Gy total) using IMRT.

Acute toxicity was evaluated weekly during radiotherapy (RT), at 1–3 months afterwards using RTOG acute scoring system. Late side effects were scored at 6, 9, 12, 16, 20, 24 and 36 months after RT using RTOG/EORTC criteria.

Quality of life was assessed by EORTC-C30 questionnaire and PR25 prostate module. Biochemical failure was defined using ASTRO consensus and nadir+2 definition, clinical failure as local, regional or distant relapse.

**Results:**

None experienced grade III-IV toxicity. 10% had no acute genito-urinary (GU) toxicity, 63% grade I; 26% grade II. Maximum acute gastrointestinal (GI) scores 0, I, II were 37%, 47% and 16%. Maximal acute toxicity was reached weeks 4–5 and resolved within 4 weeks after RT in 82%.

Grade II rectal bleeding needing coagulation had a peak incidence of 18% at 16 months after RT but is 0% at 24–36 months. One developed a urethral stricture at 2 years (grade II late GU toxicity) successfully dilated until now. QOL urinary symptom scores reached a peak incidence 1 month after RT but normalized 6 months later. Bowel symptom scores before, at 1–6 months showed similar values but rose slowly 2–3 years after RT. Nadir of sexual symptom scores was reached 1–6 months after RT but improved 2–3 years later as well as physical, cognitive and role functional scales.

Emotional, social functional scales were lowest before RT when diagnosis was given but improved later. Two years after RT global health status normalized.

**Conclusion:**

This hypofractionated IMRT schedule for PC using 25 fractions of 2.64 Gy did not result in severe acute side effects. Until now late urethral, rectal toxicities seemed acceptable as well as failure rates. Detailed analysis of QOL questionnaires resulted in the same conclusion.

## Background

Radiotherapy (RT) is one of the established primary modalities for treating prostate cancer. About 30% of all prostate cancer patients, who are treated with curative intent, receive RT [1] and a substantial proportion of these patients will be cured. The most common RT technique for treating prostate cancer is external beam radiotherapy, often delivered conformally to spare as much normal tissue as possible. A great deal of effort has been put into improving radiotherapeutic regimens for prostate cancer through brachytherapy and intensity-modulated radiotherapy (IMRT). Less attention has, however, been paid to fraction size.

Brenner and Hall [2] suggested in 1999 an α/β ratio for prostate cancer of 1.5, much lower than the typical value of 10 Gy for many other tumours and even lower than the late-responding tissues (3–4 Gy). This conclusion was based on a modelling comparison of the doses of 65–80 Gy used for external beams and the higher doses used for permanent 125-I seed implants which resulted in similar freedom from biochemical failure rates.

Recent analysis of clinical data (Fowler et al. [3]; Brenner and Martinez [4]; Bentzen et al. [5]) showed remarkable agreement with the conclusions of Brenner and Hall's 1999 paper. These estimates are consistent with the very slow proliferation characteristics of prostate tumours in comparison with other malignancies. Most prostate tumours have an extremely low proportion of cycling cells with an average potential doubling time (Tpot) before treatment of 40 days ranging from 15 to more than 60 days, compared with about 5 days for many other types of tumour [6-8].

A recent publication done by Williams et al. [9] supports the concept of a low α/β ratio but their data are more consistent with a value in the range of 2 to 5 Gy.

The disparity between the α/β value of 3–4 Gy for late complications and < 2 Gy for prostate tumours raises the prospect that we might be able to widen the therapeutic window by treating prostate cancer with hypofractionated radiation [10,11]. A similar rationale (but in the opposite direction) has worked out well in hyperfractionation for head and neck tumors [12]. In addition to possible radiobiological gains there are other benefits to a hypofractionation scheme. The shorter overall treatment time increases convenience for the patients and decreases cost. At present, the main concern is uncertainty about normal tissue toxicity of such hypofractionated protocols. So far the results and the toxicity are acceptable, but there is still a lack of long-term follow-up data.

In 12/2002 we started a phase I/II hypofractionation protocol in prostate cancer. The primary endpoint was assessment of the feasibility of a hypofractionation schedule to deliver a total dose of 66 Gy in 25 fractions of 2.64 Gy in five weeks for patients with localized prostate cancer using IMRT. Here we present our results for a group of 38 men treated between 12/2002 and 05/2006.

## Patients and methods

### Patients characteristics

From 12/2002 until 6/2005, 38 men with biopsy proven prostate adenocarcinoma and a clinically localized stage (cT1–T4 N0M0, using the UICC 2002 TNM classification) were recruited in this single institution study. Ethical committee of UZ Gasthuisberg Leuven approved the protocol and all patients provided written informed consent. WHO performance status ranged from 0–1. Mean age was 71 years (range: 54–79 years). Median pre-treatment PSA was 9.2 μg/l (range: 2.77–45.6 μg/l). Gleason scores ranged from 5 to 10. Table 1 shows the disease characteristics.

**Table 1:** disease parameters (iPSA: initial pretreatment PSA; HT: hormonal treatment).

Parameters		Number (%)
iPSA	< 10	18 (47%)
	10–20	16 (42%)
	> 20	4 (11%)
Stage	T1c	6 (16%)
	T2a	10 (26%)
	T2c	10 (26%)
	T3a	10 (26%)
	T4	2 (6%)
Gleason score	< or = 5	2 (6%)
	6–7	25 (66%)
	8–10	11 (28%)
HT	No	7 (18%)
	Yes	31 (82%)

According to the d'Amico prognostic factors 18% were low risk, 50% intermediate risk and 32% high risk patients.

31/38 patients received hormonal treatment (HT) with LHRH agonist +/- antiandrogen therapy varying from 6 months to a total of 4 years and in all cases concurrently with radiotherapy. Exclusion criteria were previous irradiation in the pelvic area, previous surgery for prostate cancer, nodal or distant metastasis proven by a CT pelvis or bone scan, presence of any psychological, familial, geographical or sociological condition potentially hampering compliance with study protocol and follow-up schedule.

### End points

Primary endpoint of the study was the occurrence of any grade II or more acute GU or GI toxicity during and within three months after RT, scored by using the RTOG scoring system. Secondary endpoints were late GU or GI toxicity scored by RTOG/EORTC scoring system; QOL with the EORTC 30 questionnaire and PR25 prostate module; biochemical free survival as defined by the 1997 American Society of Therapeutic Radiation and Oncology (ASTRO) consensus definition [13,14] and nadir + 2 definition [15,16] or clinical failure defined as local, regional or distant relapse.

### Dose and technique

All 38 patients were treated by the same hypofractionated schedule to a total dose of 66 Gy in 25 fractions in five weeks of 2.64 Gy to the prostate with 50 Gy in 25 fractions of 2 Gy to the seminal vesicles using IMRT. For late effects, characterized by an α/β of 3 Gy, this is an isoeffective schedule compared to our current schedule of 74 Gy in 37 fractions of 2 Gy. For the prostate tumor the chosen dose is equivalent to 78 Gy in 39 fractions of 2 Gy for an α/β of 1.5 Gy.

All patients were simulated in supine position with feet fixation. Skin marks representing the isocenter were placed at both sides of the hips, epigastric and at the level of the pubis. Lateral and anterior simulation X-rays were taken in order to document the position of the isocenter. Patients were instructed to empty their bladder before simulation and drink a steady amount of 250 cc water before scanning. A rectal enema was used to empty the rectum as much as possible. A CT-scan in treatment position with IV contrast with 3 mm slices taken from the anal verge to the level of the acetabulum was performed, followed by an MRI the same day. CT and MRI images were fused. Prostate, seminal vesicles and organs at risk (OAR's: bladder, rectum and anterior rectal wall) were outlined on the MRI. Rectum and anterior rectal wall were outlined from the anal verge to the rectosigmoid junction and the whole bladder was included.

The CTV1 included the prostate; CTV2 was used for the seminal vesicles. PTV1 was defined as CTV1 + 1 cm, PTV2 as CTV2 + 0.5 cm. The PTV1s were planned to receive a D99% of 59.4 Gy, D95% of 62.7 Gy, D50% of 66 Gy. The PTV2s were planned to receive a D99% of 45 Gy, D95% of 47.5 Gy, D50% of 50 Gy.

The OAR's planning limits were based on prior studies (17). Less than or equal to 25%, 50% and 70% of the rectum volume could receive respectively 70 Gy (2 Gy/fx), 45 Gy (2.64 Gy/fx), 38 Gy (2.64 Gy/fx) with a maximum tolerated dose of 76 Gy (2 Gy/fx). For the rectum the DVH's were recalculated to the equivalent dose in 2 Gy per fraction using the LQ model assuming α/β = 3 Gy and only for the dose above 50 Gy (25 fractions). Below 50 Gy, the original DVH was used as we preferred to overestimate rectal doses instead of underestimating them. Maximum dose to the anterior rectal wall was set at 66.5 Gy with a maximal dose never exceeding 13.3 Gy/week. Fifty percent of the bladder volume could receive up to 70 Gy (2 Gy/fx).

IMRT with inverse treatment planning on the Eclipse planning system (Varian) was performed using a five field 18 MV photon beam set-up. Pre-treatment verification of the dose distribution was done with an IMRT phantom and an amorphous silicon imager. During treatment the patient was advised to have a full bladder and to empty his rectum before treatment. The patient was localized daily using the BAT transabdominal ultrasound system (n = 14) or portal imaging of bony structures (n = 24).

### Toxicity

Acute side effects were scored weekly during RT, weekly afterwards until acute effects were resolved, at 1 and 3 months after RT using the RTOG scoring system. Late effects were scored at 6, 9, 12, 16, 20, 24, 36 months using the RTOG/EORTC late morbidity scoring system.

### Quality of Life (QOL)

QOL was scored at baseline; 1 and 6 months; 1, 2 and 3 years after RT using EORTC-C30 questionnaire and PR25 prostate module.

### Failure rates

Evaluation of tumour response was performed by digital rectal examination and PSA levels 3-monthly the first year after RT, 4-monthly the second and third year and from then on every 6 months until the fifth year after RT when it was on yearly basis. On suspicion of tumour recurrence or progression a CT scan of the pelvis, ultrasonography of the prostate and a bone scan were performed. Prostate biopsies were not systematically performed. We defined failure as biochemical or clinical failure. Biochemical failure was defined according to (ASTRO) consensus guidelines [13,14] as three consecutive rises in PSA level after the nadir. The nadir + 2 definition was also used as recent publications [15,16] pointed out that this definition appears to be optimal and may be selected as the new RTOG-ASTRO definition. Clinical failure included local, regional or nodal relapse and distant metastasis.

### Statistics

The Fleming one stage testing procedure was used [18]. The hypotheses were: (1–P0) is the highest probability of toxicity which, if true, implies that the irradiation schedule *does not *warrant further investigation, in this trial P0 has been taken as 50% incidence of grade II or more; (1–P1) is the lowest probability of toxicity which, if true, implies that the irradiation schedule *does *warrant further clinical investigation; in this trial P1 has been taken as 70%; α is the accepted probability of recommending for further trials the regimen if the toxicity is equal to or higher than 30%, in this trial α has been taken as 0.1; β is the accepted probability of rejecting from further trials the regimen if the stated toxicity is equal or less than 50%; in this trial β has been taken as 0.1. Under these hypotheses a total sample size of 38 patients was calculated.

## Results

Compliance with the study protocol was excellent. All 38 patients were scored according to protocol and filled in QOL questionnaires.

Median follow-up was 20 months (range 6–36) after completing RT.

### Dosimetric parameters

Table 2 shows that the mean delivered doses for the PTV1 D99%, D95%, D50% and PTV2 D99%, D95%, D50% were higher than the constraints and confirmed the RT schedule. Mean delivered doses for the OAR were lower than set up constraints.

**Table 2:** mean (range) delivered doses to PTV1 (prostate), PTV2 (seminal vesicles), OAR's (rectum, anterior rectal wall, bladder)

	**Volume**	**Constraints**	**Mean delivered dose**
**PTV1**	99%	59.4 Gy	62.2 Gy (60.7–63)
	95%	62.7 Gy	63.7 Gy (62.7–64.6)
	50%	66 Gy	66 Gy (64–66.6)
**PTV2**	99%	45 Gy	47.6 Gy (45–49.8)
	95%	47.5 Gy	49.6 Gy (47–52.3)
	50%	50 Gy	56.8 Gy (52–61)
**Rectum**	25%	70 Gy (2 Gy)	55.3 Gy (30–65.4)
	50%	45 Gy (2.64 Gy)	35.7 Gy (17–43)
	70%	38 Gy (2.64 Gy)	22.7 Gy (6.6–34.4)
	Max Dose	76 Gy (2 Gy)	74.5 Gy (66–74.9)
	Anterior rectal wall	66.5 Gy (2.64 Gy)	66.1 Gy (65–66.7)
**Bladder**	50%	70 Gy (2 Gy)	35.1 Gy (5.5–58.3)

### Acute GU symptoms (Figure 1)

**Figure 1:**
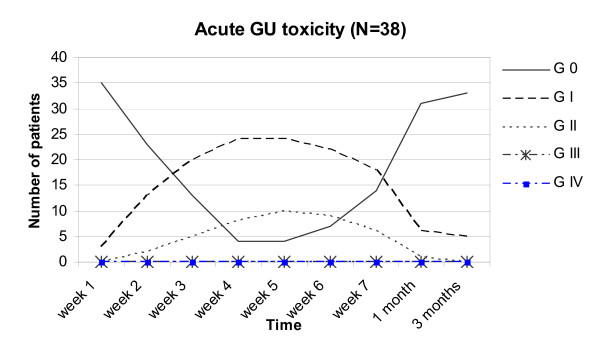
acute GU toxicity in all 38 patients.

Four patients (10%) had no acute GU toxicity while 63% (n = 24) experienced a maximum of Grade I and 26% (n = 10) Grade II during RT. None developed a grade III/IV acute GU toxicity. Acute GU toxicity reached its maximum in weeks 4 and 5 and resolved within 4 weeks after RT in 82% (n = 31) of the patients. At three months after RT, 5 patients (13%) had Grade I GU toxicity.

### Acute GI symptoms (Figure 2)

**Figure 2:**
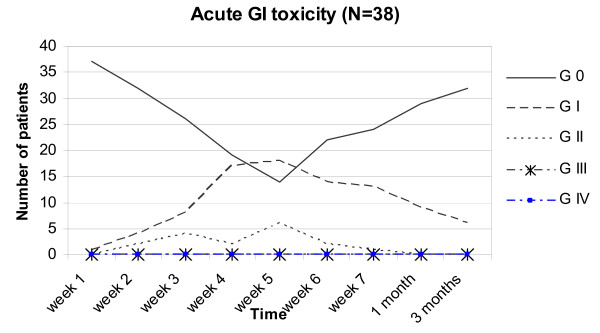
acute GI toxicity in all 38 patients.

Maximum acute GI grades of 0, I and II were respectively 37% (n = 14), 47% (n = 18) and 16% (n = 6). Detailed scoring of rectal mucus or blood loss resulted probably in the rather high incidence of Grade II toxicity. No Grade III/IV toxicity was found. At 3 months after RT, 6 men (16%) had Grade I toxicity.

### Late GU symptoms

At 6 months after RT only one (3%) patient had Grade I GU toxicity. At one year (n = 26), at 16 (n = 16), and 20 months (n = 14) after RT, none of the patients experienced GU toxicity. At two years (n = 10) one patient was diagnosed with a stricture of the urethra scored as Grade II late GU toxicity. After single dilatation dysuria disappeared. At 36 months (n = 6) no late GU toxicity was found.

### Late GI symptoms (Table 3)

**Table 3:** late GI toxicity

	6 months	9 months	12 months	16 months	20 months	24 months	36 months
N° patients	38	36	26	16	14	10	6
Grade 0	31	29	20	8	9	7	4
Grade I*	6	5	5	5	4	3	2
Grade II*	1	2	1	3	1	0	0
Grade III	0	0	0	0	0	0	0
Grade IV	0	0	0	0	0	0	0

*grade I: mucosal discharge, mild rectal bleeding not needing coagulation

*grade II: rectal bleeding with telangiectasia on rectoscopy and coagulation

At 6 months after RT, 6/38 (16%) had Grade I toxicity due to slight rectal discharge or mildly increased bowel movements. 1/38 (3%) experienced a Grade II toxicity due to intermittent rectal bleeding with rectoscopy proven telangiectasia, needing coagulation. At one year after RT 5/26 men (19%) had Grade I toxicity because of persisting slight rectal discharge, 1/26 (4%) were scored as Grade II as described above. At 16 months after RT 5/16 (31%) had Grade I toxicity; three of them because of persisting slight rectal discharge, the other two because of mild rectal bleeding. One had telangiectasia where no therapy was performed, for the other one no cause for the rectal bleeding was found. 3/16 patients (18%) complained at that time of intermittent bleeding. Telangiectasia were documented by rectoscopy and coagulation was performed with an excellent result in one patient. The other patient received a second coagulation at 20 months after RTwith a good result until now (Grade II toxicity). No Grade III toxicity was seen with a median follow up of 20 months. At 24 and 36 months there were still respectively 3/10 and 2/6 patients with Grade I toxicity due to persistent slight rectal bleeding not needing coagulation, but no Grade II or III toxicity was found.

### QOL

Following scoring procedures [19,20] we calculated the mean values of urinary, bowel and sexual symptom scales; functional scales (physical, role, emotional, cognitive and social functioning) and global health status as summarized in Table 4. Urinary symptom scores reached a peak incidence one month after RT but normalized 5 months later. They stabilized at the mean value of 4 at one, two and three years after RT which was lower than the starting value of 9.6. Bowel symptom scores before, at one and six months after RT showed a similar value of 2.5, but rose slowly to 3.1 at one year, 5 at two and 5.8 at three years after RT. A nadir of sexual symptom scores was reached from one to six months after RT but this improved to a value of 40 at two and three years after RT (compared with a value of 44 before radiation). Physical, cognitive and role functional scales showed the same pattern of a lower value at one month after RT, an increase to a maximum at one year and a slowly decrease at two and three years after RT. Emotional and social functional scales showed the lowest score before RT when diagnosis was given and improved gradually over the following months and years. Two years after RT global health status reached about the same value as before therapy. The lowest value was found at one month after RT.

**Table 4:** mean values of urinary, bowel, sexual symptom scales; functional scales; global health status

	before	1 month	6 months	1 year	2 years	3 years
N° patients	38	38	38	26	10	6
Symptom scales						
urinary	9.6	15.9	8.3	4.6	4.5	4.3
bowel	2.5	2.7	2.6	3.1	5	5.8
sexual	44	17.1	17.2	19	40	40
Functional scales						
physical	89.9	86.9	87.9	90.8	89.4	84.4
role	90.4	85.1	92.5	90.4	88.4	83.3
emotional	85.9	89.9	94.3	95.6	94.2	97.2
cognitive	87.3	86.4	88.5	89.5	81.7	77.2
social	93.5	93.6	96.5	94.9	98.4	100
Global health status	82.7	60.3	78.1	79.1	81.6	75

### Biochemical or clinical failure

At time of assessment, biochemical failure defined as three consecutive rises after the nadir according to ASTRO consensus definition occurred in 3/38 patients, one at 12 and two at 16 months posttherapy. In these three cases a 6 months course of HT was given concurrently with radiotherapy. However if nadir + 2 definition was used, no biochemical failure is reported until now. Clinical failure in terms of nodal relapse was seen in one patient one year after RT for which salvage HT was started. Unfortunately the disease became hormone refractory one year later and due to alcohol induced severe liver disorder the patient could not receive chemotherapy which resulted in death a few months later. One patient developed lung metastasis three months after RT, due to a secondary colorectal adenocarcinoma. PSA-levels of this patient are still below detection level. The latter also occurred in another patient who unfortunately died due to a metastasized lung carcinoma diagnosed 4 months after RT for prostate cancer.

## Discussion

The main objective of this study was to assess the feasibility in terms of acute genito-urinary and gastro-intestinal (primary endpoint) and late (secondary endpoint) toxicity of delivering a hypofractionated schedule of 25 fractions of 2.64 Gy to a total dose of 66 Gy in five weeks to patients with localized prostate cancer using IMRT. The hypofractionated schedule is iso-effective (at α/β = 3) for late effects with a schedule of 74 Gy (2 Gy). According to Fowler et al. [21] this hypofractionation regimen with an overall treatment time of 5 weeks and fraction number of 25 is estimated to be very unlikely to result in significantly increased late effects.

Previous late toxicity reports of radiation in prostate cancer at the Leuven University Hospital were given by Vanuytsel and Van Poppel [22] for a schedule of 2 Gy fractions to 60 Gy. They found no grade III of higher late side effects in a subset of patients prospectively randomized in EORTC trial 22911 evaluating the role of postoperative radiotherapy in pT3 patients. If the present study proved to be feasible, a multicenter phase III trial could be started comparing conventional fractionation of 74 Gy in 2 Gy fraction with hypofractionation giving 66 Gy in 25 fractions of 2.64 Gy in patients with localized prostate cancer.

Assuming a value of 3% per Gy for the slope of the tumor control probability curve, this strategy could lead to an increase in bNED from 70% at 5 years to a bNED of 82% at 5 years.

### Acute toxicity

Acute effects observed in this hypofractionated regimen were comparable to those reported by others [23-26]. A 26% grade II acute GU toxicity and 16% grade II acute GI toxicity was found with a peak incidence weeks 4 and 5 of the regimen. No grade III/IV acute GU and GI toxicity was found. This is comparable with the findings of Peeters et al. [27] in their 68–78 Gy (2 Gy) trial. Pollack et al. [28] reported in their randomized trial somewhat higher figures of grade II (40%) and grade III (8%) acute GU toxicity in the H-IMRT arm (70.2 Gy in 26 fractions of 2.7 Gy) although PTV margins were slightly smaller. Inclusion of lymph nodes in high-risk patients, the use of a modified RTOG scale and mean biological doses to the prostate exceeding 80 Gy were held responsible for these findings. Acute GI toxicity figures were similar to those reported by others. They found no statistical differences in acute GU and GI toxicity between the C-IMRT (76 Gy in 38 fractions of 2 Gy) and above mentioned H-IMRT arm. Slightly higher figures of acute GI and GU toxicity were also recently reported by Soete et al. [29] in a phase II multi-institutional study were 36 prostate cancer patients received a total dose of 56 Gy in 16 fractions over 4 weeks to the prostate. Lukka et al. [30] compared 66 Gy in 33 fractions of 2 Gy to 52.5 Gy in 20 fractions over 28 days and found higher acute urinary (5.1 vs 9.2%) and rectal (2.8 vs 4.3%) in the hypofractionated arm. Kupelian et al. [31-33] compared acute toxicity of a three-dimensional conformal radiotherapy scheme of 78 Gy in 39 fractions of 2 Gy for prostate cancer patients with a later IMRT scheme of 70 Gy in 28 fractions of 2.5 Gy. Comparable rates of acute GU (20% conformal vs 21% IMRT) and GI (19% conformal vs 14% IMRT) were found. Up until now reports on the degree of acute toxicity of prostate cancer patients treated with a hypofractionated radiotherapy regimen are not consistent, probably due to different organ at risk constraints, different radiotherapy techniques (conformal vs IMRT) and PTV margins used. Another question that needs to be answered is the influence of hormonal treatment (HT) on acute toxicity in men with prostate cancer treated with radiotherapy. Peeters et al. [34] concluded that neo-adjuvant HT appeared to be an independent prognostic factor for acute toxicity, resulting in less acute GI, but more acute GU toxicity. The first could be explained by the shrinking of the prostate and seminal vesicles with subsequent smaller RT fields and less exposure of the rectal wall [35,36]; but for the latter no obvious explanation was suggested. An additive effect of androgen suppression and external irradiation on local control by induction of apoptosis is reported by several authors [37,38]. This phenomenon could have an increasing effect on normal tissue toxicity and explain the higher acute GU toxicity rates in the hormonal therapy (HT) arm of the Peeters study. Also in our hypofractionated regimen acute toxicity figures could be influenced by this phenomenon as 31 of the 38 patients received HT concurrently with RT.

### Late toxicity

No late GU toxicity was found at 6, 9, 12, 20 months after RT. At two years one patient was diagnosed with a stricture of the urethra scored as a Grade II late GU toxicity. After dilatation his symptoms of dysuria disappeared. Late GI toxicity and especially rectal bleeding seems more important. Yeoh et al [39] reported in their randomized trial a sustained increase in GI toxicity at two years after RT compared with baseline in both arms (conventional 64 Gy in 32 fractions versus hypofractionation 55 Gy in 22 fractions). In the hypofractionated arm they found a slightly greater percentage of patients experiencing mild rectal bleeding at two years, but this difference was not statistically significant. In this study grade II rectal bleeding that needs coagulation has reached a peak incidence of 18% at 16 months after radiotherapy and is now 0% at 24 and 36 months. We believe that intensive detailed scoring for rectal bleeding followed by rectoscopy and immediate coagulation if telangiectasia was present, contributed to these figures. The majority of these patients had significant cardiac morbidity and the large use of anticoagulants could also be responsible for earlier recognition of rectal blood loss.

The influence of HT on late radiotherapy toxicity has been examined in a retrospective study by Jani et al [40]. They observed similar late GU and GI toxicity rates in 455 patients who did (n = 197) and did not (n = 248) receive HT. These findings are not consistent with other investigations that demonstrated a greater rate of late GI toxicity and especially late rectal bleeding with the use of HT. Sanguineti et al. [41] reported in a multivariate analysis 2-year estimates of grade II-IV late rectal toxicity of 30.3% in patients receiving HT versus 14% in patients without HT.

As all the present patients with grade II rectal bleeding received concurrently HT in our study, we believe that these late rectal bleeding figures could be strongly influenced by the HT addition.

### QOL

Urinary symptom scores reached a peak incidence 1 month after RT, but normalized 5 months later. At one, two and three years after RT a stabilisation was noticed and the mean value of 4 was lower than the starting value of 9.6, probably due to prostate shrinkage and tumour control. Bowel symptoms scores before, at one and six months after RT showed the same value of 2.5 but slowly rose to 3.1 at one year, 5 at two and 5.8 at three years after RT which can be explained by detailed reporting of rectal bleeding. One to six months after RT the lowest value of sexual symptoms scores was reached probably due to the concurrent use of HT with radiotherapy. Two and three years later a value of 40 (compared with the value of 44 before therapy) was found. A possible explanation for this phenomenon could be the short duration of HT in most of the patients, but also the use of 5 fosfodiësterase inhibitors especially in the younger ones. Physical, cognitive and role functional scales showed the same pattern of a lower value at one month after RT, an increase to a maximum value at one year after RT and a slow decrease at two and three years probably due to aging of the patient population. Emotional and social functional scales showed the lowest score before RT when diagnosis was given and improved gradually in the months and years after RT. Two years after RT global health status reached about the same value as before. The lowest value was also in this case reached at one month after RT.

### Failure

In one patient biochemical failure according to ASTRO consensus definition was noticed one year after RT and in two others at 16 months. A major issue is the use of a short course (6 months) of HT concurrently with RT in these cases. After cessation of HT, a transient increase in PSA may occur as a result of recovery of prostate tissue from testosterone suppression. This may lead to false-positive results with ASTRO definition and a recalculation with nadir + 2 definition was performed. With this definition no biochemical failure was seen until now. Clinical failure in terms of nodal relapse was seen in one patient one year after therapy for which salvage HT was started but resulted in death one year later.

## Conclusion

In conclusion, this phase I/II study shows acceptable acute GU and GI toxicity rates resulting from a hypofractionated regimen of 66 Gy in 25 fractions of 2.64 Gy for localized prostate cancer. Late urethral toxicity and rectal bleeding rates may be influenced by the addition of hormonal therapy but seem acceptable, although we are aware that longer follow up is needed to see if these figures can be maintained. Detailed analysis of different QOL scales resulted in the same conclusion. The future of all this is likely to include fewer and larger fractions in the radiation treatment of prostate cancer, keeping overall treatment time not too short like three or four fractions a week. The important thing, until more and more tumour results come through and we can see what α/β for tumours really is, is to keep normal tissue reactions under control.
